# Editorial: Cognitive, motor and brain reserves: bio-behavioral mechanisms, phenotypes, and prognostic value in ageing and neurodegeneration

**DOI:** 10.3389/fpsyg.2024.1502843

**Published:** 2024-11-13

**Authors:** Sonia Di Tella, Sara Isernia, Niels Bergsland, Gabriella Bottini, Annalena Venneri

**Affiliations:** ^1^Department of Psychology, Università Cattolica del Sacro Cuore, Milan, Italy; ^2^IRCCS Fondazione Don Carlo Gnocchi ONLUS, Milan, Italy; ^3^Department of Neurology, Buffalo Neuroimaging Analysis Center, School of Medicine and Biomedical Sciences, University at Buffalo, State University of New York, Buffalo, NY, United States; ^4^Department of Brain and Behavioral Sciences, University of Pavia, Pavia, Italy; ^5^NeuroMi, Milan Center for Neuroscience, Milan, Italy; ^6^Cognitive Neuropsychology Centre, ASST “Grande Ospedale Metropolitano” Niguarda, Milan, Italy; ^7^Department of Life Sciences, Brunel University London, Uxbridge, United Kingdom; ^8^Department of Medicine and Surgery, University of Parma, Parma, Italy

**Keywords:** cognitive reserve, motor reserve, brain reserve, aging, neurodegeneration, dementia

The ability to cope resiliently with age-related and neuropathological brain changes is a key scientific concern and has increasingly gained attention. The concept of reserve (Stern, 2002) has been proposed to account for the mismatch between the degree of neural insult and any detectable clinical manifestations. The most representative form of reserve is “Cognitive Reserve” (CR), defined as the brain's capacity to outperform cognitive demand against life-course brain changes, diseases, and brain insults (Stern et al., 2023). Another related emerging construct is “Motor Reserve” (MR; Sunwoo et al., 2017), which is defined as the brain's potential for enrichment and resilience to neuronal damage to sustain motor function (Giustiniani and Quartarone, 2024) thanks to mechanisms involving compensatory processes in both the brain and individual muscles. The term “Brain Reserve” (BR) has also been introduced to refer to the overall neuroanatomical resources that constitute a neural surplus supporting cognitive function despite age-related or neurodegeneration-related loss of substrate (Stern et al., 2019). This concept relies on the idea that impairment manifests only when the loss of neural substrate reaches a certain critical threshold (Satz, 1993). As a result, people at risk of neurodegeneration, but with a sufficient neural substrate buffer may be less prone to cognitive decline.

The quantification of reserve remains challenging. Proxies for CR typically rely on “sociobehavioral variables,” such as education, intelligence quotient, occupational attainment, and leisure-stimulating activities (Jones et al., 2011). Assessing MR generally consists of quantifying the engagement in both outdoor and indoor daily life activities (e.g., walking, playing sports, working, and leisure) and domestic activities that require physical effort. For BR, heterogeneous measures have been used, including both gross volumetry indexes and fine-grained measures, such as the estimated total intracranial volume, head circumference, synaptic density, and dendritic branching (Stern et al., 2019).

The relationship between brain changes, environmental/genetic factors, and brain function is complex. Environmental enrichment, such as stimulating cognitive and motor experiences accrued during life, can lead to structural brain changes and foster brain plasticity. Further understanding of the bio-behavioral mechanisms underlying reserve may prompt new approaches to prevent neural and cognitive decay. Notably, factors potentially protecting or aggravating pathophysiological processes are commonly studied when neurodegenerative insults have already occurred, often rendering it difficult to disentangle the precise contributions of cognitive and neural reserves in maintaining adequate performance (Montine et al., 2019).

The protective role of reserves has been demonstrated in clinical conditions (Garibotto et al., 2008; Cammisuli et al., 2022; Di Tella et al., 2024), with individuals having high reserve proxies showing a greater gap between brain damage and level of functioning (Isernia et al., 2024; Sunwoo et al., 2017; Chung et al., 2020).

In this article Research Topic, we present new contributions focused on factors influencing forms of reserve in healthy and neurological conditions.

In detail, Huang et al. proposed a new index, combining different neurocognitive domains, to identify conditions of subjective cognitive decline in people with Parkinson's Disease (PD) having a low level of education, who still maintain high cognitive performance even in the presence of perceived cognitive complaints. They suggested that such individuals may benefit from early management of cognitive impairment.

Investigating the relationship between lifestyle and the brain scaffold in adults with no subjective cognitive decline, Šneidere et al. found that a more intellectually and socially active lifestyle could contribute to better brain health, particularly in left temporo-parietal regions vulnerable to Alzheimer's Disease (AD). CR proxies may contribute to lowering the risk of longitudinal progression to mild cognitive impairment and AD dementia. Potential neurodegeneration, therefore, could reflect the interplay of genetic, environmental, lifestyle and other factors.

Aiming at identifying resilient neural networks, Di Tella et al. tested the hypothesis that functional connectivity alterations would be modulated by lifelong cognitively stimulating experiences. Applying two complementary methodological approaches, they focused on the main large-scale cognitive networks and key prefrontal regions associated with executive dysfunction in people with PD. In those with low CR proxies, the expression of attentional control networks appears to be reduced, whereas higher recruitment of medial frontal regions might contribute to maintaining efficient cognitive functioning.

Reserve-building research has focused on how lifelong stimulating activities can improve cognitive function and brain health not only in pathological conditions but also in healthy elderly individuals. Liang et al. studied whether maintaining good exercise habits may alleviate the decline of perceptual abilities in the course of aging. They found that elderly individuals who engaged in longer daily exercise experienced better preservation of perceptual abilities.

Schwartz et al. explored the connection between reserve and the expression of depressive symptoms that negatively impact resilience. The authors documented a beneficial effect of reserve-building activities against depression, encouraging engagement in such activities, which are highly accessible and low-cost, especially for people who are disabled, unemployed, or retired due to medical conditions.

Bernini et al. explored the role of CR proxies in identifying ideal candidates for a cognitive enhancement program in telerehabilitation for people with subjective cognitive complaints and neurocognitive disorders. Adopting a machine-learning approach, they demonstrated that CR proxies, technological skills, distance from the clinic, and age were factors able to classify patients as people willing to be involved in telerehabilitation rather than an in-clinic program.

Zhao et al. reviewed the compensation and reserve mechanisms triggered by physical exercise therapy on sleep disorders. By summarizing evidence from the past few years, the authors drew a picture of the most effective rehabilitation strategies and the molecular mechanisms involved in improving sleep disorders in people with neurological injuries after an exercise therapy program. By this approach, exercise enhancement may boost recovery in people with sleep disorders, and may constitute a factor in increasing sleep quality and duration.

The studies part of this Research Topic hint at the potential of reserve mechanism in prompting clinical decisions that should be taken into consideration both in evaluation and intervention settings, to define age-related motor and cognitive trajectories and implement personalized rehabilitation strategies.
